# Reliable and robust method for abdominal muscle mass quantification using CT/MRI: An explorative study in healthy subjects

**DOI:** 10.1371/journal.pone.0222042

**Published:** 2019-09-19

**Authors:** Jisuk Park, Jea Ryung Gil, Youngbin Shin, Sang Eun Won, Jimi Huh, Myung-Won You, Hyo Jung Park, Yu Sub Sung, Kyung Won Kim

**Affiliations:** 1 Department of Radiology, Asan Image Metrics, Asan Medical Center, University of Ulsan College of Medicine, Seoul, Korea; 2 Department of Radiology, VHS Medical Center, Seoul, Korea; 3 Department of Biomedical Engineering, Asan Medical Institute of Convergence Science and Technology, Asan Medical Center, University of Ulsan College of Medicine, Seoul, Korea; 4 Department of Radiology, Ajou University School of Medicine, Ajou University Medical Center, Suwon, Korea; 5 Department of Radiology, Kyung Hee University Hospital, Seoul, Korea; Medical University of Vienna, AUSTRIA

## Abstract

**Background:**

Quantification of abdominal muscle mass by cross-sectional imaging has been increasingly used to diagnose sarcopenia; however, the technical method for quantification has not been standardized yet. We aimed to determine an optimal method to measure the abdominal muscle area.

**Methods:**

Among 50 consecutive subjects who underwent abdominal CT and MRI for possible liver donation, total abdominal muscle area (TAMA) and total psoas muscle area (TPA) at the L3 inferior endplate level were measured by two blinded readers. Inter-scan agreement between CT and MRI and inter-reader agreement between the two readers were evaluated using intraclass correlation coefficient (ICC) and within-subject coefficient of variation (WSCV). To evaluate the effect of measurement level, one reader measured TAMA and TPA at six levels from the L2 to L4 vertebral bodies.

**Results:**

TAMA was a more reliable biomarker than TPA in terms of inter-scan agreement (ICC: 0.928 vs. 0.788 for reader 1 and 0.853 vs. 0.821 for reader 2, respectively; WSCV: 8.3% vs. 23.4% for reader 1 and 10.4% vs. 22.3% for reader 2, respectively) and inter-reader agreement (ICC: 0.986 vs. 0.886 for CT and 0.865 vs. 0.669 for MRI, respectively; WSCV: 8.2% vs. 16.0% for CT and 11.6% vs. 29.7% for MRI, respectively). In terms of the measurement level, TAMA did not differ from the L2_inf_ to L4_inf_ levels, whereas TPA increased with a decrease in measurement level.

**Conclusions:**

TAMA is a better biomarker than TPA in terms of inter-scan and inter-reader agreement and robustness to the measurement level. CT was a more reliable imaging modality than MRI. Our results support the use of TAMA measured by CT as a standard biomarker for abdominal muscle area measurement.

## Introduction

Sarcopenia is characterized by an age-related decline of muscle mass with low muscle strength and/or physical performance, and it has recently been assigned the International Classification of Diseases (ICD-10CM) code [M62.84] [1–3]. The assessment of muscle and fat tissues is essential in the management of patients with obesity, aging, and wasting diseases [4, 5]. Recent accumulating evidence strongly suggests that sarcopenia is predictive of certain clinical outcomes including postoperative complications, hospital stay, and final survival/mortality in various diseases [6–11]. Therefore, sarcopenia is regarded as a diagnostic and prognostic biomarker.

Cross-sectional imaging techniques, such as magnetic resonance imaging (MRI) and computed tomography (CT), are the most reliable methods and thus, regarded as gold standard methods for quantifying the muscle mass and visceral fat area (VFA) or volume. CT has been the most widely used cross-sectional imaging modality, because it is readily available in most hospitals worldwide owing to its reasonable cost and high scan speed. Currently, the use of MRI for the abdomen has been increasing because of radiation exposure concerns as well as the potential to achieve improved tissue contrast [12, 13].

However, the quantification of abdominal muscle mass by CT and MRI as a diagnostic biomarker for sarcopenia assessment has not been fully validated. The two main requirements for validating the abdominal muscle area as a quantitative biomarker for sarcopenia are as follows: (1) clinical validation, which involves the evidentiary process of linking the abdominal muscle area with clinical endpoints such as survival or mortality, and (2) standardization, which is the process of implementing and developing technical standards [14]. In terms of clinical validation, increasing evidence has demonstrated a strong association of the abdominal muscle area measured by cross-sectional imaging with survival of patients with various diseases such as cancer, cardiovascular disease, or trauma [15–17].

However, the technical method for quantifying abdominal muscle mass by CT/MRI has not been standardized because of the following issues: (1) The area that should be segmented on abdominal CT/MRI has not been standardized, because segmentation areas vary among total abdominal muscle area (TAMA), total psoas muscle area (TPA), and other muscle areas; (2) it is unclear whether CT and MRI are interchangeable for quantifying abdominal muscle mass because of insufficient evidence on inter-scan agreement between CT and MRI; and (3) the measurement level of abdominal muscle mass has not been standardized; it varies from L2 to L4 in prior studies [18–20].

So far, only a few studies have examined these issues [20]. Therefore, we aimed to determine an optimal method for measuring the abdominal muscle area.

## Materials and methods

This retrospective study adhered to the guidelines established by the Declaration of Helsinki, and it was approved by the Institutional Review Board of Asan Medical Center, Seoul, Korea (No. 2018–0382). The requirement for informed consent was waived.

### Patients

We retrospectively searched our institution’s computerized databases for a clinical cohort of liver transplantation and found 50 consecutive healthy subjects who underwent abdominal CT and MRI for possible liver donation from March 2016 to June 2016. All the liver donors underwent CT and MRI within a 2-week interval as a preoperative work-up for liver transplantation.

CT and MR images were anonymized and transferred from our picture archiving and communication system (PetaVision; Hyundai Information Technology, Seoul, Korea) to the central imaging review system (AiCRO^TM^; Asan Image Metrics, Seoul, Korea). A staff (S.E.W.) of an imaging core lab in our institution (Asan Image Metrics, www.aim-aicro.com) independently performed the imaging process following our request.

### Image acquisition

#### Computed tomography

The CT examinations were performed using a Somatom Definition AS+ scanner (Siemens Healthineers, Erlangen, Germany). The CT examinations were obtained with standard exposure parameters (200 effective mAs and 120 kVp; the actual radiation dose was adjusted according to the patient’s body size and shape by automatically modulating the tube current, a detector configuration of 1.5 mm × 16 mm, a table feed of 24 mm per rotation, and a gantry rotation time of 0.5 s. Contrast-enhanced CT scans were performed in the supine position in the portal venous phase with a fixed delay of 70 s after contrast agent injection. By using an autoinjector, 120 mL of nonionic contrast material was intravenously administered at the rate of 3 mL/s. The images were reconstructed with a section thickness and interval of 5 mm.

#### Magnetic resonance imaging

Abdominal MRI was performed using a 1.5T scanner (Magnetom Avanto; Siemens Healthineers, Erlangen, Germany) with dedicated six-channel torso array coils. The maximum gradient strengths were 45 mT/m for the amplitude and 200 mT/m/s for the slew rate. The parameters for the transverse breath-hold T1-weighted gradient-echo images without fat-suppression were as follows: repetition time, 4.2 ms; echo time, 2.5 ms; flip angle, 7.0°; slice thickness, 3 mm; field of view, 341 × 420 mm; and matrix size, 208 × 256. These images were used for body morphometric analysis. The other imaging sequences of the abdominal MRI included transverse T2-weighted fast spin-echo imaging, MR cholangiography, in-phase and opposed-phase chemical shift imaging, and contrast-enhanced multiphasic MRI.

#### Body morphometric analysis

Our imaging processing team members (Y.S., S.E.W., and Y.S.S.) developed AsanJ-Morphometry^TM^, a software dedicated to the measurement of abdominal muscle and fat area, on the basis of ImageJ (NIH, Bethesda, MD, USA). The software is publicly available for non-profit research in a website (available at http://datasharing.aim-aicro.com/en/morphometry).

**Fig 1:**
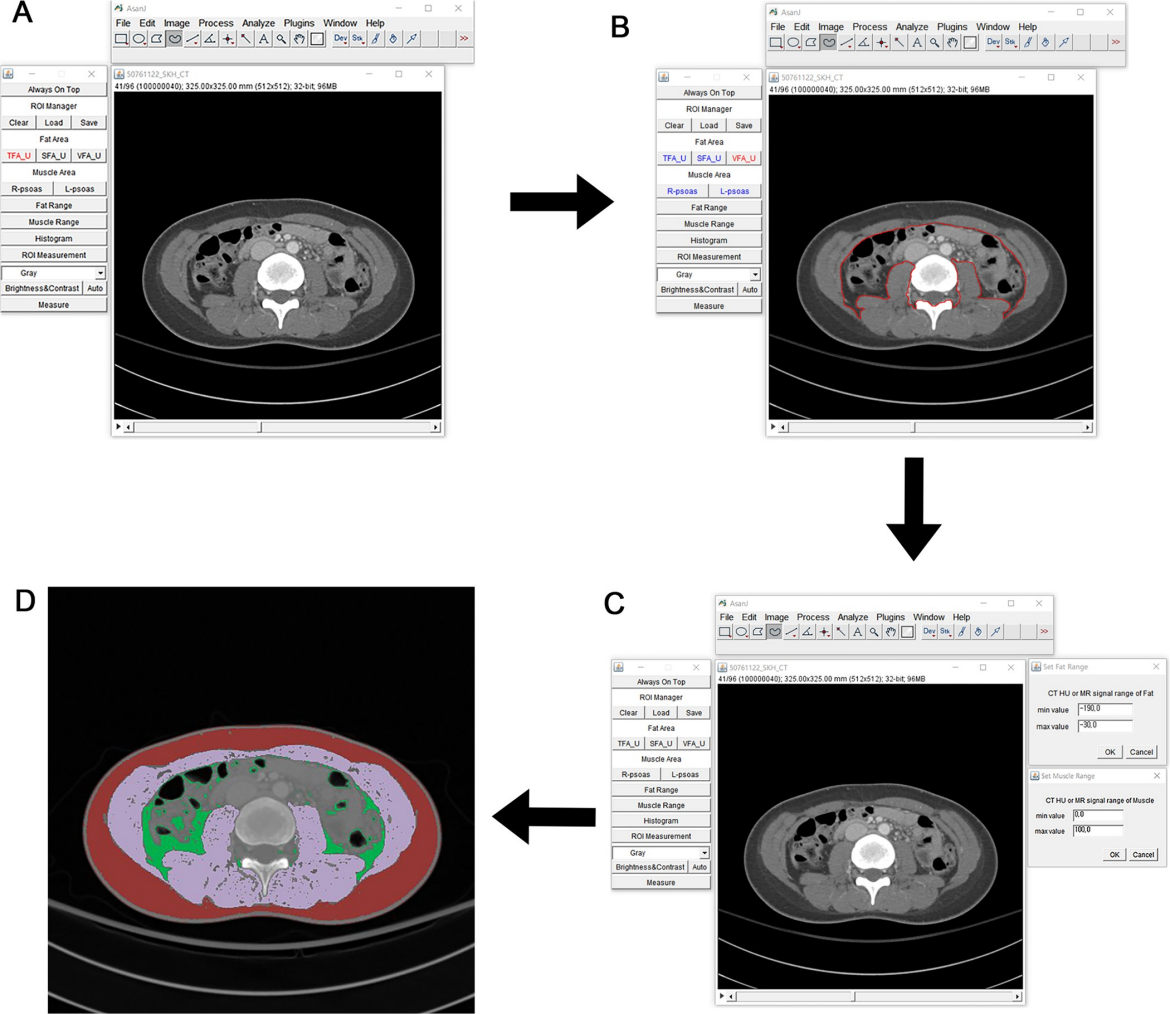
Semi-automatic segmentation for body morphometric analysis. (A) ImageJ after running the program and uploading a CT or an MR image. (B) Adjustment of the threshold of total abdominal muscle area (TAMA), total psoas muscle area (TPA), and visceral fat area (VFA). (C) Measurement of the semi-automatic regions of interest (ROI). (D) Segmentation and color mapping.

TAMA (cm^2^) including all the muscles on the selected axial images, i.e., psoas, paraspinal, transversus abdominis, rectus abdominis, quadratus lumborum, and internal and external obliques, was demarcated using predetermined thresholds for the Hounsfield unit (HU) on CT (>−30 HU and ≤150 HU) and the signal intensity (SI) on precontrast T1-weighted MRI (>350 SI and ≤750 SI). The VFA (cm^2^) and subcutaneous fat area (SFA) (cm^2^) were also demarcated using the fat tissue thresholds in CT (≤−30) and MRI (>100 SI and ≤350 SI) (Fig 1).

To evaluate the inter-reader and inter-scan agreements, we selected CT and MR images at the L3 inferior endplate level. Reader 1 (J.P., image analyst with 11 years experience) and reader 2 (J.R.G., abdominal radiologist with 5 years experience) independently measured TAMA, VFA, SFA with blinding to clinical information. The time spent on measuring TAMA, TPA, and VFA using the AsanJ-Morphometry^TM^ software was recorded only by a reader (J.P.). The definition of the time spent was set to include opening the software, importing the prepared CT/MR images, finding the L3 inferior endplate level, and segmenting the abdominal muscle.

### Level of body morphometric measurement

**Fig 2:**
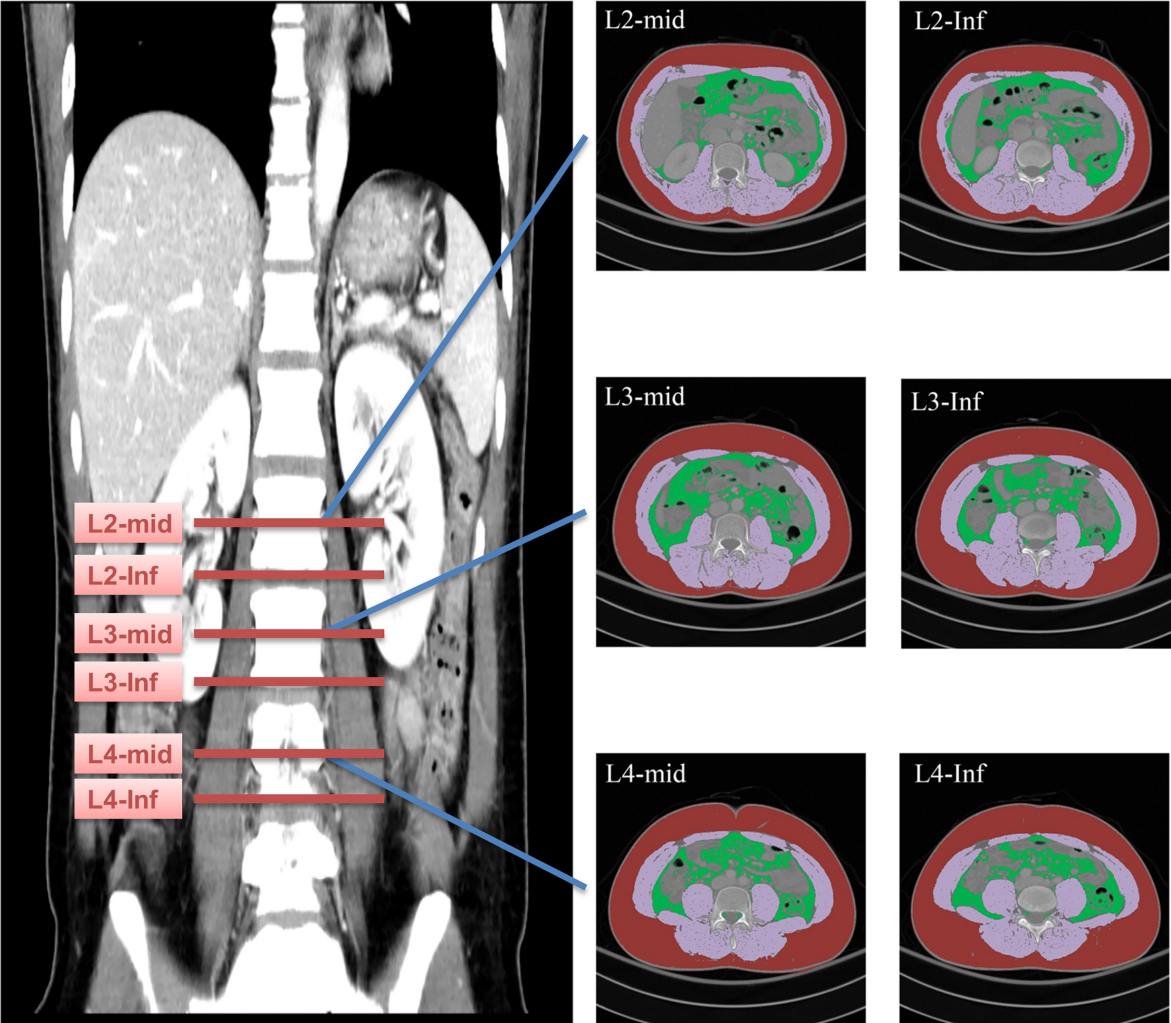
Representative images of TAMA, TPA, and VFA at different measurement levels. The vertebral bodies were measured at different vertebral levels. The axial CT images were segmented into TAMA (purple), TPA (blue), and VFA (green).

To evaluate the effect of measurement level on the results of body morphometric analysis, an abdominal radiologist (J.H., 9 years experience) measured TAMA, TPA, and VFA at six levels starting from the L2 to L4 vertebral bodies (Fig 2). For each vertebral body level, we performed measurements at the mid-body level (hereafter referred to as L2_mid_, L3_mid_, and L4_mid_) and inferior endplate level (hereafter referred to as L2_inf_, L3_inf_, and L4_inf_).

### Statistical analysis

Data are expressed as mean ± standard deviation (SD). The mean values of body morphometric analysis were compared by Student’s *t*-test or analysis of variance (ANOVA) and post-hoc multiple comparison tests.

The measurement agreements between CT and MRI (inter-scan agreement) and between readers 1 and 2 (inter-reader agreement) were assessed on the basis of the intraclass correlation coefficient (ICC) of a single measurement calculated according to the two-way random-effects model, for consistency. The 95% confidence intervals (CIs) associated with the ICCs were also determined. The ICC estimates the overall correlation between all possible values within the variable taken by the same reader. The ICCs were interpreted as poor (0.00–0.49), fair (0.50–0.74), and good (0.75–1.00) [21].

To evaluate the inter-reader and inter-scan agreements, we used statistical tools recommended by the methodological guidelines of the Radiological Society of North America-Quantitative Imaging Biomarkers Alliance (RSNA-QIBA) (https://www.rsna.org/QIBA) and Park et al. [14–16]. By using these methods, the within-subject coefficient of variation (WSCV) and repeatability coefficient (RC) were calculated. Bland-Altman plots were also constructed. To evaluate the difference between measurement levels, one-way ANOVA was performed with post-hoc Tukey-Kramer pairwise comparison tests. For statistical analysis, we used a web-calculator (available at http://datasharing.aim-aicro.com/reliability) and MedCalc version 13.1.2 (MedCalc Software, Ostend, Belgium).

## Results

### Patients

The average age (mean ± SD) of the 50 subjects was 29.9 ± 8.3 years (range, 17–57 years; median, 37; interquartile range, 11.5). The subjects included 29 men (mean age, 29.03 years; range, 18–57 years) and 21 women (mean age, 31.05 years; range, 17–47 years).

### Body morphometric analysis

The measured TAMA, TPA, and VFA values are summarized in Table 1. There was no significant difference between reader 1 and reader 2 with regard to the measurement of TAMA (*t*-test, P = 0.925 for CT, P = 0.121 for MRI), TPA (P = 0.738 for CT, P = 0.223 for MRI), and VFA (P = 0.919 for CT, P = 0.01). Similarly, no significant difference was observed between CT and MRI with regard to the measurement of TAMA (*t*-test, P = 0.333 for reader 1, P = 0.636 for reader 2), TPA (P = 0.520 for reader 1, P = 0.097 for reader 2), and VFA (P = 0.154 for reader 1, P = 0.176 for reader 2).

**Table 1:** Mean TAMA, TPA, and VFA values derived by body morphometric analysis.

	TAMA (cm^2^)			TPA (cm^2^)			VFA (cm^2^)		
	CT	MRI	P^a^	CT	MRI	P^a^	CT	MRI	P^a^
**Reader 1**	137.0 ± 37.9 (137.6, 59.1)	144.6 ± 40.6 (139.0, 59.9)	0.333	18.6 ± 8.7 (17.5, 12.3)	17.4 ± 9.5 (14.6, 11.6)	0.520	67.6 ± 44.0 (57.8, 60.4)	62.0 ± 42.3 (51.1, 54.3)	0.516
**Reader 2**	136.3 ± 38.0 (139.8, 59.8)	132.8 ± 34.6 (127.2, 52.7)	0.636	18.0 ± 8.7 (16.8, 12.7)	14.7 ± 6.5 (14.1, 11.0)	0.097	68.5 ± 45.2 (60.5, 64.0)	56.5 ± 42.3 (44.6, 48.6)	0.176
**P**^b^	0.925	0.121		0.738	0.223		0.919	0.525	

Data are presented as mean ± standard deviation. Data in parentheses represent the median value and interquartile range.

^a^Derived by comparing CT and MRI using *t*-test

^b^Derived by comparing reader 1 and reader 2 using *t*-test

Abbreviations: TAMA = total abdominal muscle area; TPA = total psoas muscle area; VFA = visceral fat area

By using the ImageJ-based software (AsanJ-Morphometry^TM^), the mean time spent by reader 1 on measuring TAMA, TPA, and VFA was 3.63 ± 0.57 min for CT and 5.65 ± 1.55 min for MRI (P < 0.001, *t*-test). The time spent for MRI was longer, which may be attributed to a greater difficulty in identifying the L3 inferior endplate level on MR images and the necessity of adjusting the semi-automatically drawn muscle boundaries.

### Inter-scan and inter-reader agreements

The ICC, WSCV, and RC for inter-scan and inter-reader agreements are shown in Table 2. Bland-Altman plots for all pairs of comparison are illustrated in the Supporting Information S1, S2, and S3 Figs.

**Table 2:** Inter-scan and inter-reader agreements for TAMA, TPA, and VFA.

		Inter-scan agreement		
		ICC	WSCV (%)	RC
**TAMA**	**Reader 1**	0.928	8.3	32.484
	**Reader 2**	0.853	10.4	38.811
**TPA**	**Reader 1**	0.788	23.4	11.694
	**Reader 2**	0.821	22.3	10.341
**VFA**	**Reader 1**	0.950	15.9	28.576
	**Reader 2**	0.899	25.9	44.850
		**Inter-reader agreement**		
**TAMA**	**CT**	0.986	8.2	31.010
	**MRI**	0.865	11.6	44.448
**TPA**	**CT**	0.886	16.0	8.1217
	**MRI**	0.669	29.7	13.499
**VFA**	**CT**	0.989	6.9	13.011
	**MRI**	0.954	16.6	27.169

TAMA = total abdominal muscle area; TPA = total psoas muscle area; VFA = visceral fat area; ICC = intraclass correlation coefficient; WSCV = within-subject coefficient of variation; RC = repeatability coefficient

The comparison of the inter-scan and inter-reader agreements for TAMA, TPA, and VFA yielded ICC values that were generally higher than 0.75. However, the ICC of TAMA was higher than that of TPA with regard to both inter-scan agreement (0.928 vs. 0.788 for reader 1 and 0.853 vs. 0.821 for reader 2, respectively) and inter-reader agreement (0.986 vs. 0.886 for CT and 0.865 vs. 0.669 for MRI, respectively). Based on the WSCV, the reliability of TAMA was better than that of TPA with regard to both inter-scan agreement (8.3% vs. 23.4% for reader 1 and 10.4% vs. 22.3% for reader 2, respectively) and inter-reader agreement (8.2% vs. 16.0% for CT and 11.6% vs. 29.7% for MRI, respectively). These findings suggest that the measurement of TAMA might be a more reliable method for abdominal muscle mass quantification than the measurement of TPA.

**Fig 3:**
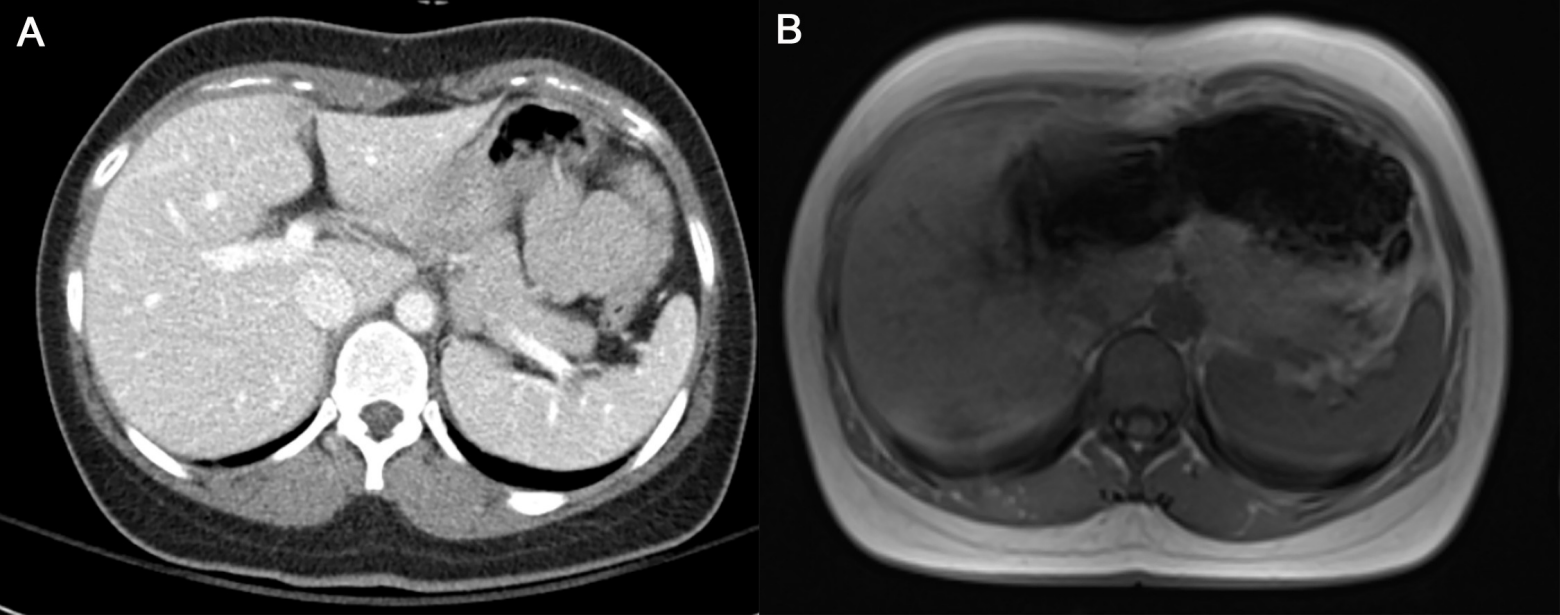
Quality of CT and MR images for TAMA measurement. (A) In the CT image, the abdominal muscle boundary is clear. (B) In the MR image, the presence of susceptibility and motion artifacts due to bowel gas degraded the image quality of the adjacent left rectus muscle and left psoas muscle.

A comparison of the inter-reader agreement between CT and MRI revealed that the ICC for CT was higher than that for MRI in the measurement of TAMA (0.986 vs. 0.865), TPA (0.886 vs. 0.669), and VFA (0.989 vs. 0.954). The WSCV for CT was also lower (i.e., better reliability) than that for MRI in the measurement of TAMA (8.2% vs. 11.6%), TPA (16.0% vs. 29.7%), and VFA (6.9% vs. 16.6%). We evaluated all regions of interest (ROI) on CT and MR images in a side-by-side manner and found that the anatomical boundary of the muscles was degraded and less clear in some parts because of artifacts such as bowel gas susceptibility artifacts, motion artifacts, or chemical shift artifacts (14/50, 28%) (Fig 3).

### Effect of measurement level on body morphometric analysis

**Fig 4:**
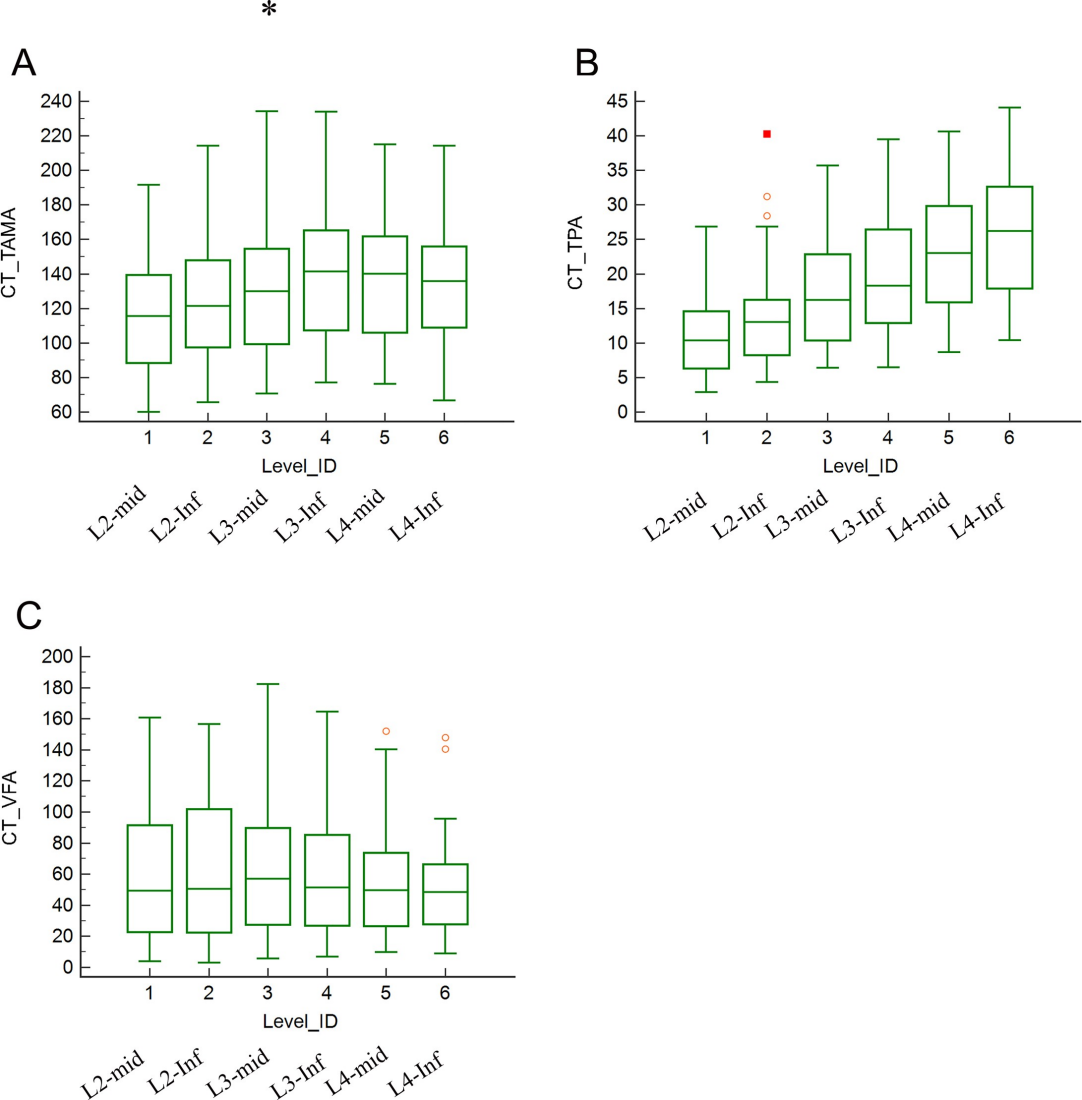
Effect of measurement level on TAMA, TPA, and VFA. (A) TAMA at L2_mid_ was different from that between L2_inf_ and L4_inf_, and it was similar at other levels. (B) TPA was different at every level. (C) VFA was not significantly different between levels.

One-way ANOVA revealed a significant difference between measurement levels in TAMA (P = 0.003) and TPA (P < 0.001) but not in VFA (P = 0.525). The post-hoc test results for TAMA showed a significant difference only between L2_mid_ and L3_inf_ (P < 0.05) and between L2_mid_ and L4_mid_ (P < 0.05). The TAMA from L2_inf_ to L4_inf_ did not differ significantly (range, 122.5–139.6 cm^2^) (Fig 4A). The mean TAMA values of L3_mid_ and L3_inf_, which are the most commonly used measurement levels, were 132.3 ± 36.1 cm^2^ and 139.6 ± 36.6 cm^2^, with no significant difference shown by post-hoc test (P > 0.05). In contrast, TPA increased with a decrease in measurement level from L2_mid_ to L4_inf_; significant differences were noted between levels (Fig 4B). VFA did not differ among the measurement levels (Fig 4C). According to the results, the measurement of TAMA and VFA was robust to measurement level from L2_inf_ to L4_inf_. The raw data is provided in the Supporting Information S1 File.

## Discussion

This study aimed to standardize a method for the quantification of abdominal mass using CT/MRI by demonstrating that TAMA is more reliable and robust than TPA in terms of inter-scan agreement and inter-reader agreement as well as the effect of measurement level. The results also indicated that the measurement of TAMA can be easily integrated into routine clinical care by using a software, which is highly reliable in quantifying body composition from clinically acquired CT/MRI scans.

We investigated why TAMA is more reliable than TPA in terms of the inter-scan and inter-reader agreements and found that readers may have difficulty in manually drawing the posterior margin of the psoas muscle on both CT and MR images, because the psoas muscle is closely attached posteriorly to the quadratus lumborum and erector spinae muscle. In contrast, TAMA is generally calculated by a semi-automatic software on the basis of predetermined thresholds of the HU in CT or SI in MRI.

In terms of measurement level, TAMA was different between the L2_mid_ and L2_inf_–L4_inf_ levels and similar between the L2_inf_ and L4_inf_ levels. Therefore, TAMA can be measured anywhere between the L2_inf_ and L4_inf_ levels. However, TPA was generally larger at a lower measurement level; consequently, TPA was different between the L2, L3, and L4 levels. Therefore, when using TPA as an index, it is important to select one level and measure at the same level consistently. As TAMA is less affected by the measurement level, it is considered as a more robust index of abdominal muscle mass than TPA.

Contrary to our initial expectation, CT was more robust and reliable for abdominal muscle mass quantification than MRI based on the ICC and WSCV values. This may be attributed to a clearer anatomical boundary of the muscles on CT images than on MR images. Bowel gas and motion artifacts caused the degradation of image quality of the adjacent abdominal muscle; thus, the readers faced difficulty in drawing the boundaries of the muscle. Based on the results, the measurement of TAMA by CT might be the most robust method for sarcopenia evaluation compared to the measurement of TAMA by MRI and that of the TPA by CT/MRI.

Recently, studies on sarcopenia have been rapidly increasing because of various potential clinical applications such as the mortality assessment of patients requiring liver transplant [22], selective and non-abdominal aortic aneurysm repair [23, 24], and pancreatic adenocarcinoma treatment [25] and elderly patients requiring emergency surgery [26, 27].

In particular, patients with cancer are vulnerable to muscle wasting and they easily fall into a cachectic state; thus, sarcopenia assessment plays an important role. As most of the patients with cancer are followed up by CT/MRI, there are increasing efforts to evaluate the muscle mass using CT/MRI scans [27–29]. In many studies, TPA was used mainly because it is easier and faster to measure than TAMA [25, 30]. Nevertheless, the results in our study showed that TAMA is more robust than TPA; TPA was higher at a lower level, and the posterior margin was not well distinguished, which is a disadvantage in terms of reliability. Indeed, our findings are consistent with those of several prior studies [6, 31]. In addition, TAMA has been shown to be a valid surrogate marker of the whole body muscle mass because it reflects all muscles of the abdomen [29, 32].The results of our study would support the results of these prior studies by adding the value of reliability of TAMA.

Only one study has compared imaging modalities for sarcopenia assessment, which demonstrated that TAMA measured at the L3 level was comparable between CT and MRI for patients with liver cirrhosis [33]. In contrast, our study showed that CT was more robust than MRI. Differences in the imaging protocol or measurement software may cause these inconsistencies; thus, further studies are required.

Currently, measurement methods and measurement levels have not been standardized. On the basis of our results, we propose that TAMA rather than TPA should be used to reliably quantify the abdominal muscle mass. If possible, CT should be the primary cross-sectional imaging modality. However, if only MRI is available, then measurement by MRI would be acceptable. Regarding the measurement level, L3 level has been widely used, because the muscle mass measured on L3 level reflects the whole body muscle mass well [34]. Most of the studies used L3_mid_ level, where transverse processes are fully visualized [35, 36], while some used L3_inf_ level nearest the inferior aspect of vertebral body [37, 38]. In our study, there was no significant difference in the measurements between L3_mid_ and L3_inf_, and both levels may represent L3 level. To standardize the measurement level, further large-scale studies and international consensus meeting would be necessary.

Body morphometric analysis based on cross-sectional images can be easily integrated into routine clinical care by using a simple image processing software to perform reliable measurement of the abdominal muscle and fat with clinically obtained scans. As increasing evidence supports cross-sectional imaging-based surveillance as an objective method for identifying sarcopenia in patients with various diseases, clinically acquired CT/MRI scans of patients with various diseases may be used concurrently to diagnose sarcopenia, identify patients at risk of poor survival, and contribute towards general health improvement [15].

There are several limitations to this study. First, this study was conducted in a retrospective manner with a relatively small number of subjects. A large-scale, prospective validation study is needed. Second, the subjects enrolled in this study were healthy prospective liver donors, which might limit the generalizability of the study results. Nevertheless, it was the best approach to accumulate data for the measurement of abdominal muscle area while minimizing the confounding effects of pathological conditions. This method should be further evaluated using patients with various diseases. Third, among various MRI sequences, we measured abdominal muscle and fat quantity only in the T1-weighted image without fat-saturation. The value of abdominal muscle mass quantification in the other MRI sequences would be our future research topic.

In conclusion, as a cross-sectional imaging-based biomarker of sarcopenia, TAMA was more reliable than TPA in terms of inter-scan and inter-reader agreements and robustness in measurement. Furthermore, CT was a more reliable imaging modality than MRI. To use these sarcopenia biomarkers in clinical practice, the standard measurement methods should be determined from the international consensus of academic communities on the basis of large-scale evidence obtained from both healthy subjects of variable age ranges (young adults to elderly subjects) and patients with various diseases.

## Supporting information

S1 Fig Bland-Altman plots for TAMA. (A) CT vs. MRI for reader 1 (Inter-scan agreement). (B) CT vs. MRI for reader 2 (Inter-scan agreement). (C) Reader 1 vs. Reader 2 for CT (Inter-reader agreement). (D) Reader 1 vs. Reader 2 for MRI (Inter-reader agreement).
(TIF)

S2 Fig Bland-Altman plots for TPA. (A) CT vs. MRI for reader 1 (Inter-scan agreement). (B) CT vs. MRI for reader 2 (Inter-scan agreement). (C) Reader 1 vs. Reader 2 for CT (Inter-reader agreement). (D) Reader 1 vs. Reader 2 for MRI (Inter-reader agreement).
(TIF)

S3 Fig Bland-Altman plots for VFA. (A) CT vs. MRI for reader 1 (Inter-scan agreement). (B) CT vs. MRI for reader 2 (Inter-scan agreement). (C) Reader 1 vs. Reader 2 for CT (Inter-reader agreement). (D) Reader 1 vs. Reader 2 for MRI (Inter-reader agreement).
(TIF)

S1 File The raw data of the body morphometry measurement values. (XLSX)
