# Supplementary figures and images for: Reliable and robust method for abdominal muscle mass quantification using CT/MRI: An explorative study in healthy subjects
Source: PLoS One. 2019 Sep 19;14(9):e0222042. doi: 10.1371/journal.pone.0222042 (PMC6752777; doi:10.1371/journal.pone.0222042)

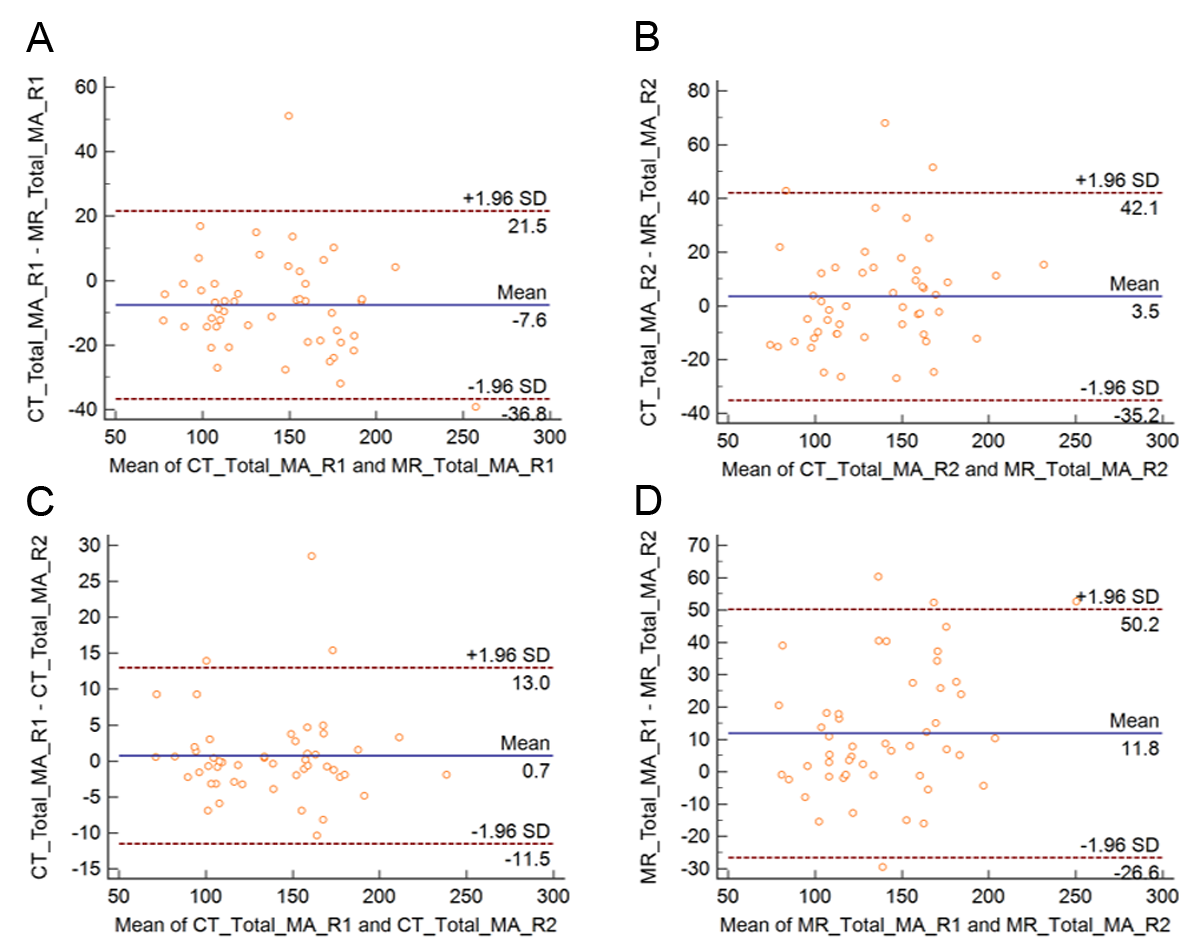

Supplement: S1 Fig — (A) CT vs. MRI for reader 1 (Inter-scan agreement). (B) CT vs. MRI for reader 2 (Inter-scan agreement). (C) Reader 1 vs. Reader 2 for CT (Inter-reader agreement). (D) Reader 1 vs. Reader 2 for MRI (Inter-reader agreement). (TIF) [file pone.0222042.s001.tif]

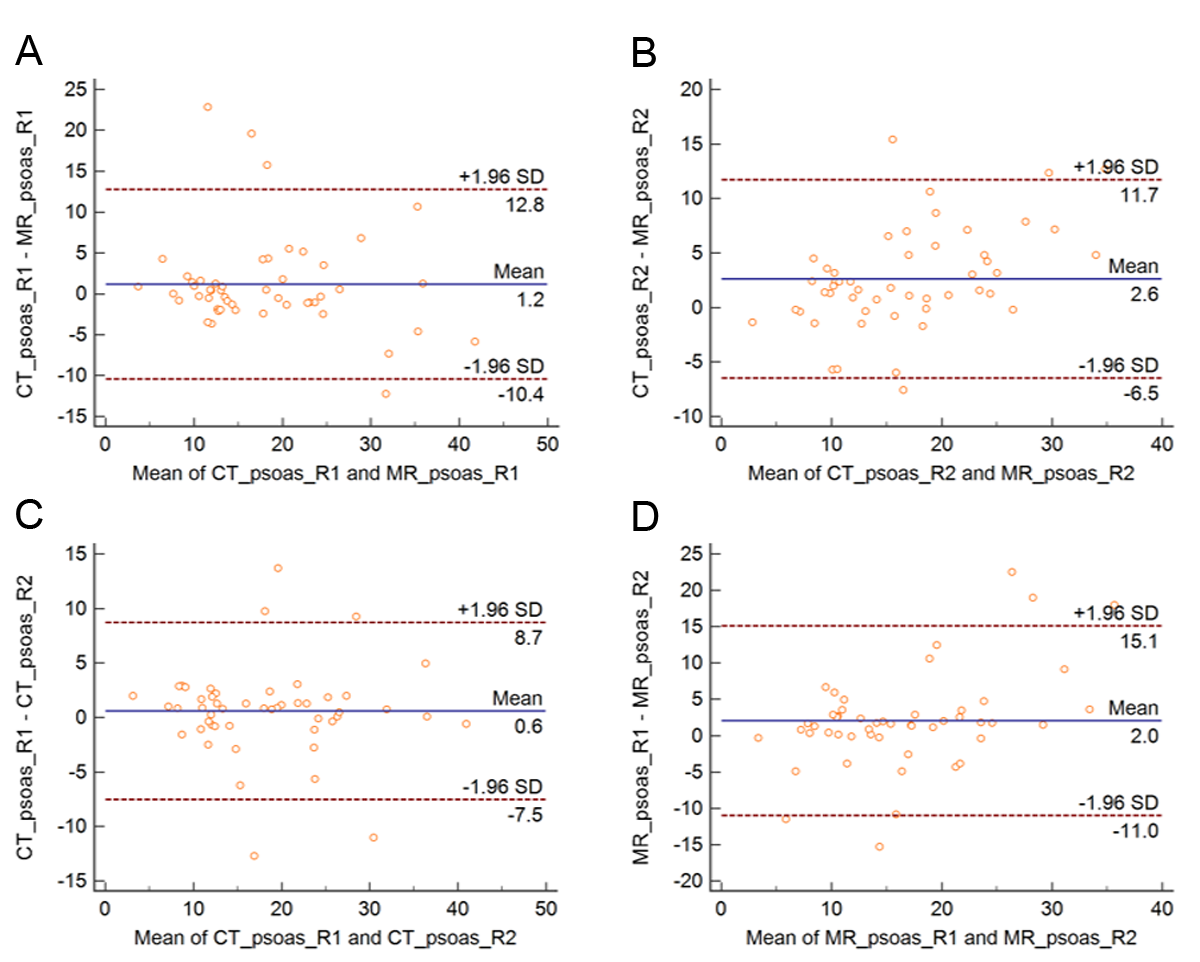

Supplement: S2 Fig — (A) CT vs. MRI for reader 1 (Inter-scan agreement). (B) CT vs. MRI for reader 2 (Inter-scan agreement). (C) Reader 1 vs. Reader 2 for CT (Inter-reader agreement). (D) Reader 1 vs. Reader 2 for MRI (Inter-reader agreement). (TIF) [file pone.0222042.s002.tif]

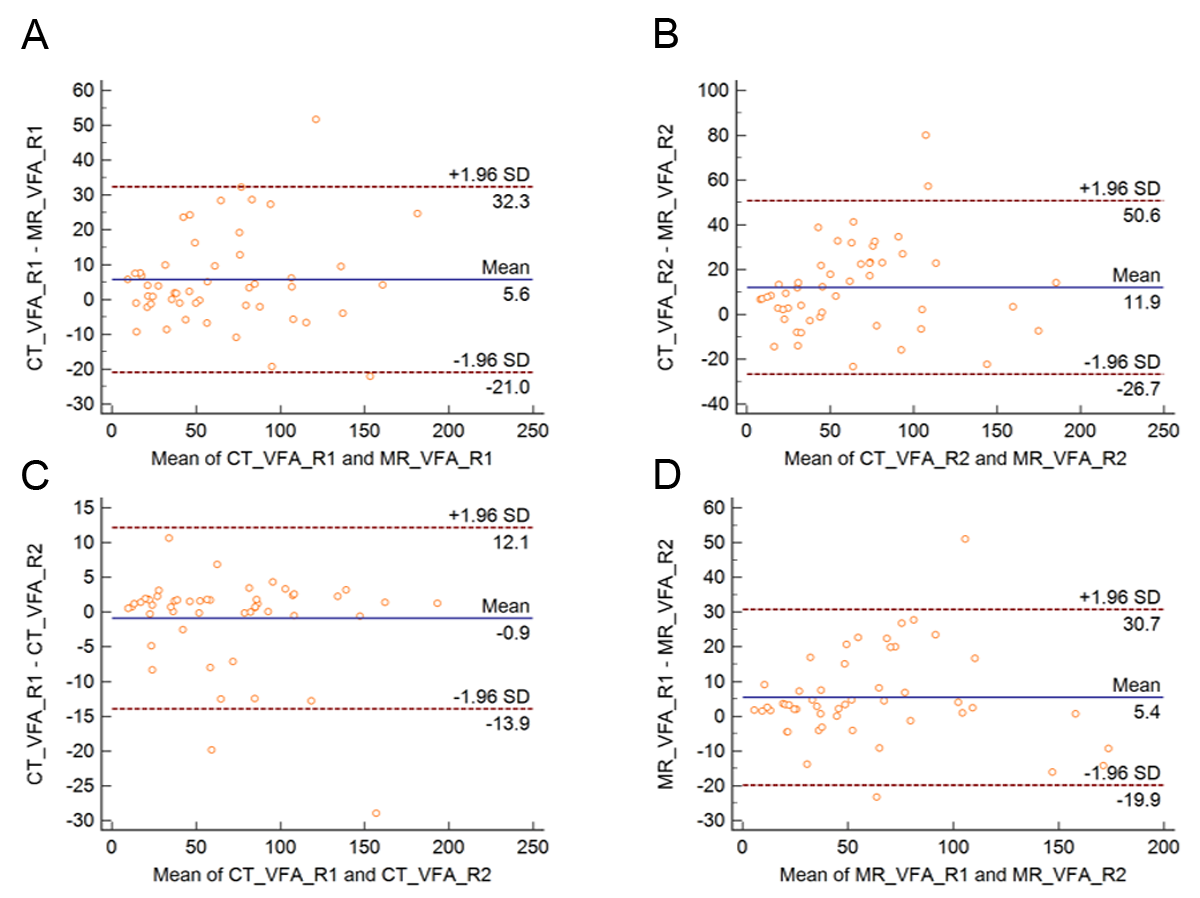

Supplement: S3 Fig — (A) CT vs. MRI for reader 1 (Inter-scan agreement). (B) CT vs. MRI for reader 2 (Inter-scan agreement). (C) Reader 1 vs. Reader 2 for CT (Inter-reader agreement). (D) Reader 1 vs. Reader 2 for MRI (Inter-reader agreement). (TIF) [file pone.0222042.s003.tif]
